# Sensing Dynamically Evolved Short‐Range Nanomechanical Forces in Fast‐Mutating Single Viral Spike Proteins

**DOI:** 10.1002/smsc.202300029

**Published:** 2023-06-11

**Authors:** Amir Farokh Payam, Riccardo Funari, Gaetano Scamarcio, Nikhil Bhalla

**Affiliations:** ^1^ Nanotechnology and Integrated Bioengineering Centre (NIBEC) School of Engineering Ulster University York Street Belfast, Northern Ireland, BT15 1AP UK; ^2^ Healthcare Technology Hub School of Engineering Ulster University York Street Belfast, Northern Ireland, BT15 1AP UK; ^3^ Dipartimento Interateno di Fisica “M. Merlin” Università degli studi di Bari Aldo Moro via Orabona 4 70126 Bari Italy; ^4^ Institute for Photonics and Nanotechnologies (IFN) CNR via Orabona 4 70126 Bari Italy

**Keywords:** Hamakar constant, nanomechanics, single-proteins, viruses

## Abstract

Understanding changes in the mechanical features of a single protein from a mutated virus while establishing its relation to the point mutations is critical in developing new inhibitory routes to tackle the uncontrollable spread of the virus. Addressing this, herein, the chemomechanical features of a single spike protein are quantified from alpha, beta, and gamma variants of SARS‐CoV‐2. Integrated amplitude‐modulation atomic force microscopy is used with dynamic force–distance curve (FDC) spectroscopy, in combination with theoretical models, to quantify Young's modulus, stiffness, adhesion forces, van der Waals forces, and the dissipative energy of single spike proteins. These obtained nanomechanical properties can be correlated with mutations in the individual proteins. Therefore, this work opens new possibilities to understand how the mechanical properties of a single spike protein relate to the viral functions. Additionally, single‐protein nanomechanical experiments enable a variety of applications that, collectively, may build up a new portfolio of understanding protein biochemistry during the evolution of viruses.

## Graphical abstract

The interplay of mechanical features of viral proteins with biochemical features of the protein is elucidated. Detection of short‐range interaction forces in viral mutants is achieved using a combination of imaging and force spectroscopy.

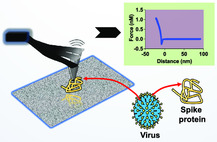

## 1 Introduction

Understanding the connection between mechanical properties and the function of a single protein molecule is the key to uncover new functional mechanisms in cell biology.^[^ ^1^, ^2^ ^]^ Apart from rendering structural features to a cell, the mechanical properties such as the adhesion and stiffness of a single protein play a crucial role in the transport/storage of small molecules and executing chemical reactions within a cell alongside the cell‐to‐cell interaction.^[^ ^3^, ^4^ ^]^ Moreover, unveiling the mechanical properties of membrane proteins in single‐cell organisms and viruses is crucial for understanding several mechanisms associated with their life cycle. For example, in the case of SARS‐CoV‐2 infection, it is well‐known that the trimer crown‐shaped spike protein (protein S) is involved in both receptor recognition, binding, and cell membrane fusion process into the host cells.^[^ ^5^, ^6^, ^7^ ^]^ Briefly, the SARS‐CoV‐2 virus infects the cell by a mechanochemical process in which the spike protein is activated proteolytically at the S1/S2 boundary (S1 and S2 being the subunits of the spike protein). In this mechanism, the S1 subunit mediates host recognition while the S2 subunit experiences a significant structural change and drives the membrane fusion between the virion and the host cell.^[^ ^7^ ^]^ Through this entry process, the lysosomal protease, cathepsin, and the surface protease TMPRSS2 are activated,^[^ ^1^ ^]^ initiating further downstream processes associated with the COVID‐19 disease. Therefore, understanding/establishing the correlations between structure, function, mutation, and mechanical properties of the S protein is of paramount importance to develop new inhibitors for blocking infection mechanism at cellular level, support vaccine development, and optimize therapeutic strategies.

Recently, several researchers have used simulation or advanced microscopy techniques to reveal the structure of the spike protein of the SARS‐CoV‐2 virus, including its mutants.^[^ ^8^, ^9^, ^10^, ^11^, ^12^ ^]^ The mechanical properties of the whole virus as well as its molecular interaction and binding process have also been characterized in some recent works.^[^ ^13^, ^14^, ^15^, ^16^, ^17^, ^18^, ^19^ ^]^ However, simultaneous extraction and quantification of nanoscale mechanical properties of a single spike protein while establishing a relationship to virus mutation are nontrivial and remain elusive. Underpinning reasons for this are associated with the lack or complexity of robust and accurate quantitative methodologies to link the measurable experimental data to the parameters used for characterizing nanomechanical properties of the single proteins. The amplitude modulation atomic force microscopy (AM‐AFM) provides the measure of the oscillation amplitude and phase shifts of a cantilever kept in semicontact oscillatory motion close to a surface. The analysis of these shifts gives access to interaction force and energy dissipation, thus enabling experimental observables to be linked with chemomechanical properties (such as viscoelasticity, stiffness, and adhesion force/energy) of a given biomolecule. This unique feature opens the path for quantitative nanoscale spectroscopy.^[^ ^20^, ^21^, ^22^ ^]^ Here, we have employed AM‐AFM and single protein spectroscopy (SPS) techniques to fully characterize the mechanical properties of three major variants being monitored (VBMs) of SARS‐CoV‐2, alpha (B.1.1.7), beta (B.1.351), and gamma (P.1).^[^ ^18^, ^23^, ^24^, ^25^ ^]^ First, using the dynamic amplitude modulation AFM, we provide the topographical characterization of the three proteins. Thereafter, we perform SPS via dynamic force curve (FDC) approach to reconstruct the interactive force profile between the AFM tip and the individual proteins. To these force profiles, we used our own developed theoretical models^[^ ^26^, ^27^, ^28^ ^]^ to extract both mechanical and chemical properties (i.e., Young's modulus, stiffness, Hamaker constant, adhesion force, and dissipation). Finally, we discuss these properties, both quantitatively and qualitatively, while associating them with mutation‐related fast‐evolving features of the virus.

## 2 Results and Discussion

In AM‐AFM mode, the cantilever is excited at or close to its natural resonance frequency, and the feedback controller is used to keep the vibration amplitude of the cantilever at setpoint value. The topographic information is acquired from the feedback loop while the phase difference between excitation and the detected cantilever signals provides information about compositional features and dissipative properties of specimen.^[^ ^28^ ^]^

**Figure 1:**
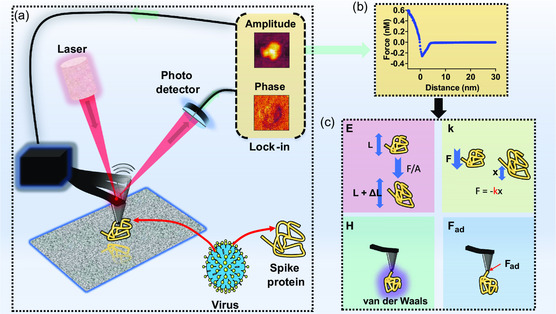
Sensing scheme. a) The oscillating cantilever of the atomic force microscope in the process of measurement of amplitude and phase changes associated with the imaging of the spike protein of the virus; b) the force versus distance curve generated following the “approach and retract” motion of cantilever from the specimen; and c) the mechanical features, which are extracted from force versus distance curve. These include Young's modulus (*E*), stiffness (*k*), Hamaker constant (*H*), and adhesion force (*F*_ad_). Within the schematic of Young's modulus, *L* and Δ*L* reference to the length and change in length of the protein.

The dynamic force–distance curve is generated by following the “approach and retract” movement of the cantilever from the sample surface while AFM operates in the amplitude modulation mode. Two variables, associated with the aforementioned observables, amplitude (*A*) and phase (*ϕ*) are acquired as a function of piezo displacement which is subsequently converted to distance between tip and sample. Then, based on our proposed force reconstruction method,^[^ ^26^ ^]^ the captured amplitude/phase versus distance (APD) curves are converted to the force versus distance profiles. Subsequently, the chemical and mechanical parameters involved in the interaction including Young's modulus, stiffness, Hamaker constant, and adhesion force are extracted from the force profiles and AM‐AFM observables.^[^ ^27^, ^29^, ^30^ ^]^ Note that the protein surfaces are imaged both before and after performing the force spectroscopy. This allows selecting, with sub‐nanometric precision, the locations to probe by force spectroscopy while avoiding any significant drift during conducting the spectroscopy. The schematic of the measurement system is shown in **Figure** **1**.

**Figure 2:**
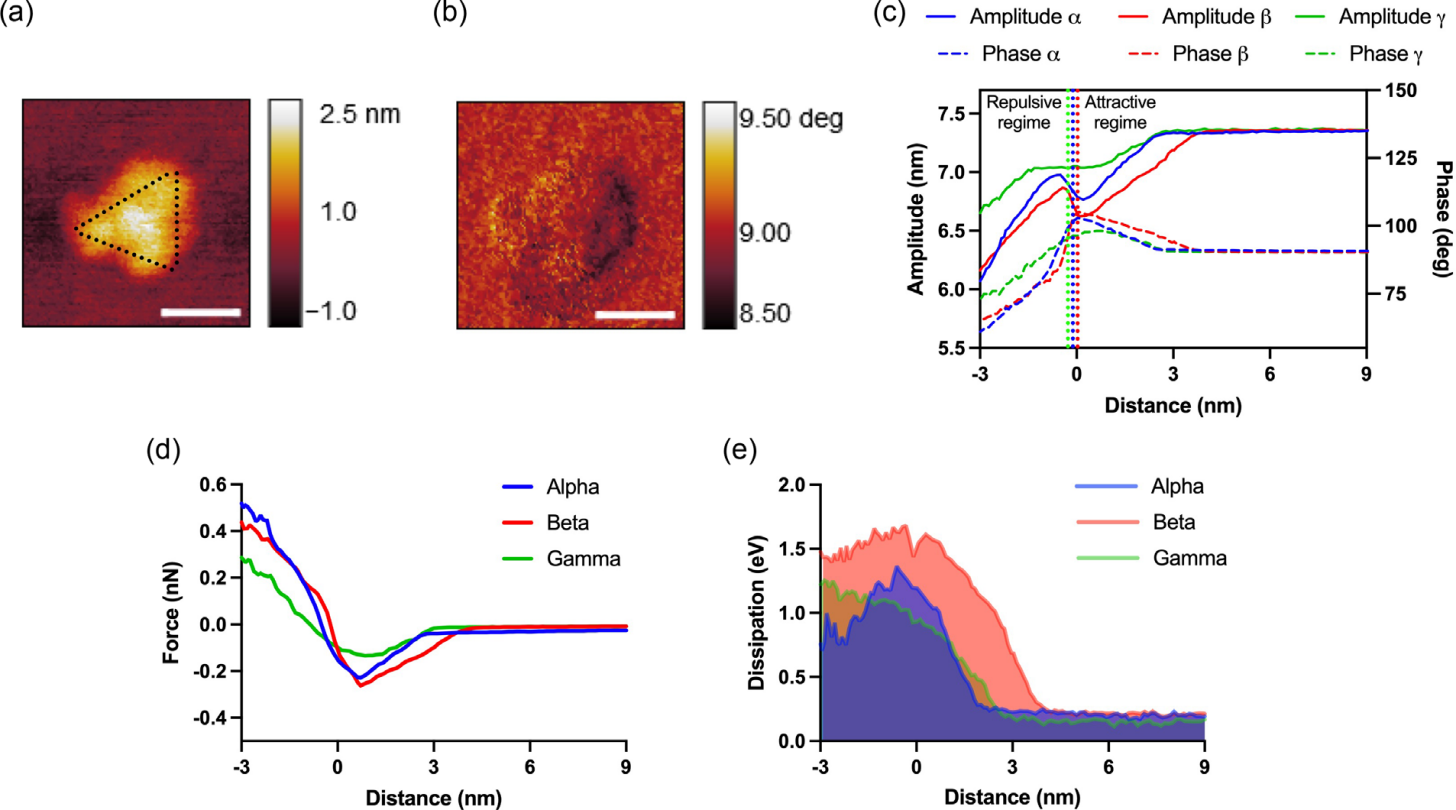
Physiochemical characterization of spike proteins by single protein spectroscopy. a) A single spike protein with a trimeric shape (the horizontal bar is 30 nm). The dotted line is a guide to an eye showing the trimer shape. b) Phase differences of the topographic image in (a). c) Representative amplitude/phase versus tip‐surface distance of AFM response of alpha, beta, and gamma proteins. The vertical dotted lines indicate the transition from attractive force regime to a repulsive force regime as shown in the figure. d) Force versus distance plot showing the difference in the interaction of AFM tip with three different proteins. e) Dissipation versus distance plot.

The representative results of acquired AFM images, amplitude/phase versus distance curves, reconstructed interaction forces between AFM tip, and proteins surfaces as well as dissipated energy of proteins are given in **Figure** **2**a–e. Since the excitation frequency in AM‐AFM is fixed, the amplitude and the phase lag of the oscillation provide two channels to explore tip–surface conservative (elastic) and dissipative (inelastic) interactions. At large distances between the AFM tip and the proteins surface, the amplitude is insensitive to the distance variations. This is followed by a region where the amplitude begins to reduce by decreasing the average tip‐surface separation which can be attributed to the interaction existing between tip and surface. More specifically, this interaction can be associated to the electrostatic, van der Waals, hydrophobic forces, chemical adhesion, or short‐range versus long‐range interactions.^[^ ^31^ ^]^ As shown in Figure 2c, the amplitude curves are characterized by the presence of a local maximum which can be attributed to the competition between attractive and repulsive interaction regimes.

The transition for gamma is smoother than alpha and beta proteins, which can be correlated to lower values of attractive and repulsive forces in comparison with the two other proteins. The phase profiles in Figure 2c, show a similar physical trend. Essentially, decreasing the tip–sample separation leads to attractive interaction as the phase starts to increase more than 90°, which is an indication of the attractive regime. This happens first for beta followed by alpha and gamma proteins. After reaching the maximum peak, the phase starts to decrease which is expected in the intermittent contact mode. Here again, the transition for gamma is smoother than that for alpha and beta which confirms our hypothesis that gamma has lower interactive forces compared to alpha and beta. In addition, within this region, the AFM probe begins to contact the surface, and due to the Pauli principle, there would be repulsion between atoms of the AFM tip and molecules/atoms of proteins. Coinciding the phase profiles with amplitude curves indicates the transition between an initial region where the amplitude is reduced by the action of long‐range attractive forces (the contact time is equal to zero) to another region where the amplitude reduction is dominated by short‐range repulsive forces (with a very short contact time). To reconstruct the interaction forces, we used the following equation^[^ ^26^ ^]^

(1) $$F_{c}\left(d\right)=2k\int_{d}^{\infty }Xdx+2k\int_{d}^{\infty }\frac{\sqrt{A}}{8\sqrt{\pi \left(x-d\right)}}Xdx-2k\frac{\partial }{\partial d}\int_{d}^{\infty }\frac{A^{\frac{3}{2}}}{\sqrt{2\left(x-d\right)}}Xdx\;$$

where *k* is the spring constant of the cantilever, *A* is the amplitude of the cantilever, *d* is the closest distance between tip and surface, and *X* is defined by

(2) $$X=\frac{A_{0}}{2QA}\text{cos}\phi$$

where *ϕ* is the phase, $A_{0}$ is the free amplitude, and *Q* is the quality factor.

The reconstructed forces of representative amplitude/phase profiles (Figure 2c) are shown in Figure 2d. From the reconstructed forces, it is clear that the interaction between tip and protein molecules begins at larger distance values for beta with respect to alpha and gamma, respectively. This finding can be explained by the larger electrostatic force for the Beta variant, where its mutations have proven to reduce the binding of antibodies.^[^ ^32^, ^33^ ^]^ In Figure 2d, the value of maximum attractive forces for beta (0.24 nN) is slightly higher than alpha (0.21 nN) while for gamma (0.12 nN) it is reduced by ≈50%. This can be explained by the higher contribution of van der Waals, hydrophobic, or chemical adhesion in alpha and beta than gamma. Furthermore, the force profiles are clearly attributed to the amplitude and phase behavior in which at the initial region of interaction between tip and surface, not only the average force but also its slope is negative (noncontact region). Additionally, in the transition between noncontacts to intermittent contact regions, the average force is negative whereas its slope corresponds to a positive value. Subsequently, by indenting the surface, which can be described by fully repulsive regime, both the average force and its slope would be positive.

The energy dissipation is calculated by the following equation^[^ ^34^ ^]^

(3) $$E_{\text{diss}}=\frac{\pi kA^{2}}{Q}\left(\frac{A_{0}}{A}\text{sin}\phi -\frac{\omega }{\omega_{0}}\right)$$

where *ω* and $\omega_{0}$ are the excitation and resonance frequencies of the cantilever in rad s^−1^, respectively.

In AFM analysis, the energy dissipation occurs at the atomic and molecular level and is described by specific atomic processes in the interaction between tip and sample including surface energy hysteresis, viscoelasticity, intramolecular charge transfer, long‐range dissipative interfacial interactions, as well as moving mirror charges at large separation of the tip from the sample. In Figure 2e, we plot the energy dissipated on the proteins surface as a function of the distance while the tip approaches the surface. Generally, the energy dissipated in the interaction between the AFM tip and the spike proteins is around 0.95–1.5 eV higher for beta, followed by gamma and alpha. Consistent with the force profile, the dissipation for beta begins to increase earlier than alpha and gamma which can be associated to the long‐range dissipative interfacial interactions and moving mirror charges caused by electrostatic forces. In the attractive regime, the dissipation of alpha is higher than gamma while in the intermittent contact/repulsive regime, the dissipation of gamma is increased and is more than alpha. Furthermore, the maximum dissipation for beta and alpha occurs at the attractive regime and decreases as the tip goes to indent the surface while for gamma there is an increase of dissipation from attractive to repulsive regimes. Therefore, it can be summarized that the shape of the dynamic dissipation profile for gamma is dominated by sample deformation which can be correlated to surface energy hysteresis while for alpha and beta, besides that effect, the long‐range dissipative interfacial interactions contribute significantly which can be related to the charges caused by electrostatic force.^[^ ^35^ ^]^

**Figure 3:**
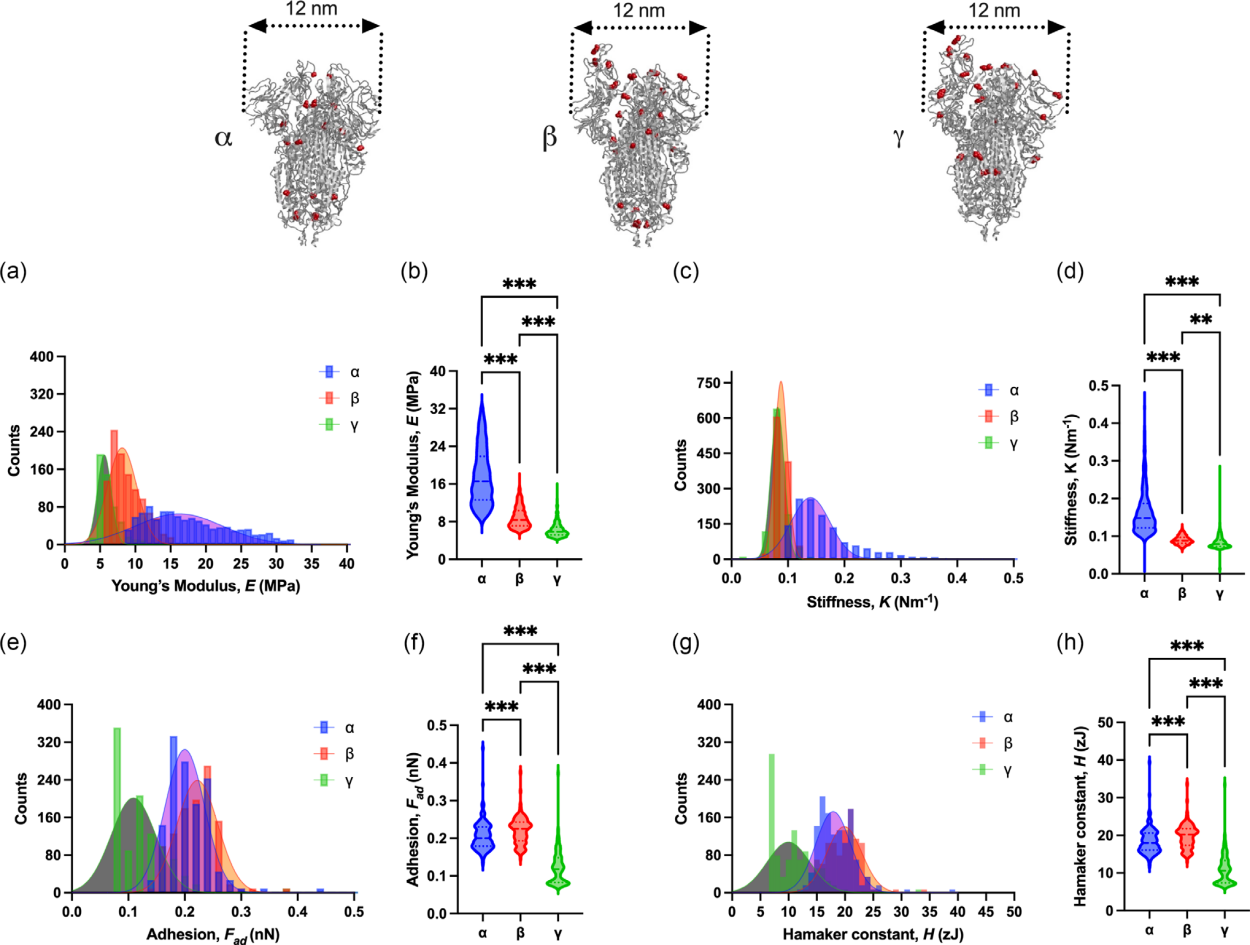
Mechanical characterization of spike proteins by single protein spectroscopy. a,b) Young's modulus distribution and the statistical comparison of Young modulus between alpha, beta, and gamma proteins. c,d) Stiffness and the statistical comparison of stiffness between alpha, beta, and gamma proteins. e,f ) Variations in the adhesive force among the three proteins. g,h) Hamaker constant and comparison of Hamaker constant of alpha, beta and gamma proteins, respectively.

The results for quantification of mechanical features of the proteins are shown in **Figure** **3**. We extract Young's modulus, *E* (MPa), stiffness, *k* (N m^−1^) adhesion force, *F*_ad_ (nN), and effective Hamaker constant, *H* (zJ) of proteins (the Hamaker constant between antimony‐doped Si probe and proteins in air environment) from reconstructed forces and AM‐AFM parameters/observables (more details about the quantification approach are given in the supporting information and data analysis sections). The obtained Young's modulus of spike protein fall in the range of 5–35 MPa in good agreement with the findings for similar proteins^[^ ^29^, ^36^ ^]^ (see Figure 3a,b). It is also worth mentioning that experimentally there is no reference to the spike S‐protein Young's modulus, to the best of our knowledge. From the comparison between Young's modulus of the three variants, it is revealed that the alpha variant is the most rigid followed by beta and gamma (see Figure 3b). The differences in the *E* values suggest that gamma should be the softest protein, while alpha should be the stiffest protein among alpha, beta, and gamma. This is validated by our obtained stiffness values, see Figure 3c,d, which also show a trend supporting the physical relevance associated with values extracted for *E* in Figure 3a,b. Moreover, the obtained stiffness values are consistent with the range reported by Kiss et al.^[^ ^14^ ^]^ One possible explanation for the larger stiffness of alpha may be attributed to the new noncovalent interactions involving the amino acids introduced in the mutations, including N501Y, A570D, P681H, T716I, S982A, and D1118H, which increase the interaction force of the protein in repulsive regime (in contact with receptors).^[^ ^37^ ^]^ Additionally, there might be several other mechanical features, which contribute to large stiffness, which is a subject for future exploration.^[^ ^38^, ^39^, ^40^ ^]^ Furthermore, our finding is consistent with the results from surface plasmon resonance (SPR) analysis regarding comparison of molecular interactions between alpha, beta, and gamma proteins.^[^ ^25^ ^]^ The calculated adhesion forces are given in Figure 3e,f. Our findings are in excellent agreement with the reported values for spike proteins adhesion forces^[^ ^14^, ^16^ ^]^ measured using AFM. Generally, this adhesion force (adhesion to the AFM cantilever tip) in spike proteins can be related to either nonspecific interactions including hydrophobic and van der Waals forces or specific interactions dominated by electrostatic force.

As expected, the beta (0.22 nN ± 0.03) has higher mean adhesion force followed by alpha (0.20 ± 0.04 nN) and then gamma protein (0.11 ± 0.04 nN). The higher adhesion of alpha and beta in comparison to gamma might be due to the fact that N501 is the key residue in alpha and beta.^[^ ^41^, ^42^ ^]^ While this may have effects on the binding of the spike protein to the host cell, we do not associate any biological activity of proteins with the host cell here. For example, in literature, it has been reported that N501Y mutation improves the binding affinity and favorable cation π stacking interactions of RBD.^[^ ^43^, ^44^, ^45^ ^]^

Finally, we extract the effective Hamaker constant of proteins using our proposed method:^[^ ^27^ ^]^

(4) $$H=-\frac{3kA_{0}A^{2}\text{cos}\phi }{QR}{\left({\left(\frac{d+A}{A}\right)}^{2}-1\right)}^{3/2}$$

where *R* is the tip radius. The Hamaker constant is indicative of van der Waals forces which contribute to the interactions of proteins with other molecules or with surfaces in contact. Moreover, the van der Waals force is a crucial factor that contributes to the formation of protein–ligand complexes. Therefore, knowing the Hamaker constant of proteins can be a key factor in predicting the molecular binding of protein–ligand or protein–protein pairs.^[^ ^46^ ^]^ Our results show similar mean values for the Hamaker constant of alpha (18.5 zJ) and beta (19.75 zJ), while lower value for gamma (10.94 zJ). Note that here we do not associate the calculated values with any direct molecular binding of spike proteins with cell receptors. These values only show comparative differences in the Hamaker constant of proteins of virus mutants.

Though new exclusive studies for correlating mechanical properties with the biological activity of viral proteins are necessary in the future, our finding can be complementary to the existing physical understanding and knowledge, explaining why although gamma variant has some of the same mutations in its spike protein as the alpha and beta strains, and still it has different biological activity as compared to the alpha and beta variants.^[^ ^32^ ^]^ More qualitative and statistical analyses regarding three different variants are given in supplementary information. Some structural differences between the spike proteins characterized using small‐angle X‐ray scattering are also available in our recently published work.^[^ ^47^ ^]^ Additionally, we would also like to mention that the behavior of virus and infectivity are complex and cannot be entirely explained by the properties of a single protein.

## 3 Conclusion

Based on the integration of imaging and spectroscopy modes of AM‐AFM and the developed quantitative methodologies, we explore the nanomechanical and chemical properties of three different VBM variants of SARS‐CoV‐2 spike protein. Our finding underlying the mechanism of energy dissipation in spike proteins explains the major contributing atomic processes. Furthermore, due to the long‐range interfacial interaction, beta has 40% higher dissipation (between 1.5 and 1.57 eV) in comparison to the alpha and gamma proteins. We quantify Young's modulus of all spike proteins in the range of 5–35 MPa which is similar to Young's modulus of amino acid‐based entities of similar molecular weights.^[^ ^36^ ^]^ In contrast, alpha is the stiffest variant in the VBM category. We find that the adhesion force of beta and alpha are close to each other in magnitude while for gamma, it is lower than beta and alpha. This can also provide physical evidence for same degree of stability of alpha and beta variants.^[^ ^48^ ^]^ A comparison between the attractive and repulsive behavior of alpha and beta reveals that the repulsive (attractive) interaction for alpha (beta) strain is more dominant than beta (alpha) variant. Finally, our findings can pave the way to analyze the folding, flexibility, and stability of several other proteins based on the exploration of their nanomechanical properties. For instance, as AFM can capture real‐time images of protein molecules as they undergo conformational changes, combining our method with well‐established AFM imaging modes which can help researchers to understand the dynamics of protein folding and perhaps understand how it is influenced by factors such as temperature, pH, and the presence of ligands. Moreover, the correlation between the mechanical properties of the proteins involved in the infection process and viral biochemical features, such as binding affinities, viral replication rates, or immunogenicity, can in principle provide a better understanding of the properties of the pathogens and pave the way for new biomedical investigation methods.

## 4 Experimental Section

4.1

4.1.1

##### Method of Sample Preparation

Alpha, beta, and gamma proteins were purchased from antibodies‐online.com: Alpha, SARS‐CoV‐2 Spike protein lineage B.1.1.7, product number: ABIN6963738; Beta, SARS‐CoV‐2 Spike protein lineage B.1.351, product number: ABIN6963739; Gamma, SARS‐CoV‐2 Spike protein lineage P.1, product number: ABIN6964442, For analysis, protein samples were prepared by incubating fresh cleaved mica with spike protein solutions (5 μg mL^−1^ in HEPES buffer) for about 90 s. Subsequently, the solution is recovered with a pipette, and the mica sheet was rinsed with abundant MilliQ water (Millipore) to wash away weakly bonded proteins. The sample was then dried with nitrogen and glued onto a magnetic AFM sample holder. All the samples were prepared 30 min before the scanning. These experimental conditions prevent the formation of protein clusters and allow to effectively disperse the biomolecules onto the mica surface to perform single protein imaging.

##### AFM Measurements and Single Molecule Spectroscopy

AFM measurements were performed in air, at room temperature, in tapping mode using an NTEGRA (NT‐MDT, Russia) microscope equipped with a gold back‐coated, antimony‐doped Si cantilever from NT‐MDT (NSG01) with typical values of a nominal tip radius of ≈6 nm, spring constant *k* ≈ 5.1 N m^−1^, and resonance frequency ≈150 kHz. To precisely control the position of the AFM probe over the protein sample, piezo scanners were calibrated at the start of each experimental session. This was performed by applying a saw‐tooth voltage (±300 mV) to the *X*‐, *Y*‐ or *Z*‐ sections of the scanner and measuring the capacitance displacement over the three different directions. This is performed before each experimental session, and correction is eventually introduced to ensure the linearity of the piezo response. The precise spring constants of the cantilevers were measured using the thermal tuning method. The areas of 400 × 400 nm were imaged under a scanning speed of 0.5 Hz, with the resolution of 512 pixels line^−1^, and relatively high amplitude setpoint ratio (*A*_sp_/*A*_free _≈ 0.9). Both topography and phase images were processed using Gwyddion software (v. 2.61). After scanning the 400 × 400 nm areas and identifying the single proteins on the mica surface, based on amplitude modulation force spectroscopy, amplitude–distance, and phase–distance‐curves are collected. This is performed with ramp rate of 0.5 Hz, ramp distance of 120 nm (this corresponds to a rate of ≈60 nm s^−1^), and resolution of 1,000 points per line.

##### Data Analysis

The collected amplitude–distance and phase–distance curves are converted to the force using our developed methodology (Equation (1)) explained in Payam et al.^[^ ^26^ ^]^ We apply the force reconstruction method for every single protein. As the tip diameter is comparable with the protein size, we located the tip on the top of the protein, and we collected several force curves. In this case, we have excluded the curves which show significant differences in their slope since the mica substrate, on which proteins are absorbed, is harder than proteins. For our analysis, we have used seven sets of measurements consisting of 77 amplitude/phase curves for alpha proteins, six sets of measurements including 95 curves for beta proteins, and six sets of measurements including 84 curves for gamma proteins. Each force–distance curve (FDC) consists of more than 2,000 points. For force reconstruction, we wrote a program in a MATLAB (MathWorks, Natick, MA, USA) environment. Then, based on the methodology explained in the article and Supplementary Information, we extract Young's modulus, Hamaker constant, dissipation, and stiffness parameters from each force curve. For this purpose, as the measurements are performed in an air environment, the point of the maximum value of phase where the phase is more than 90° is considered as a contact point. We use the contact point to separate the attractive and repulsive regimes and determine the origin of indentation. After pretreatment, to extract the Hamaker constant, we use the attractive region of the curves and to calculate Young's modulus and stiffness, we concentrate on the indentation curves. The dissipation is calculated from Equation (3) using the measured values of amplitude/phase. We use Equation (4) to calculate the Hamaker constant. To calculate Young's modulus and stiffness, for the case of spherical probes, the indentation curves are fitted using the Hertz model, while for sharp probes the Sneddon model was used.^[^ ^49^ ^]^ It is worth to mention due to the small thickness of proteins, there is a possibility of substrate effect or even touching the substrate during indentation which leads to the change of the amplitude/phase curves slope, so in our analysis, we consider these effects.^[^ ^29^, ^50^ ^]^ Moreover, to consider the effect of adhesion on the calculated Young's modulus, we use our developed method presented in the study of Payam et al.^[^ ^27^ ^]^ All of the analysis has been performed in MATLAB using a written program.

## Conflict of Interest

There are no conflicts to declare.

## Supporting information

Supplementary Material

## Data Availability

The data that support the findings of this study are available from the corresponding author upon reasonable request.
