# Supplementary material for: Sensing Dynamically Evolved Short‐Range Nanomechanical Forces in Fast‐Mutating Single Viral Spike Proteins
Source: Small Sci. 2023 Jun 11;3(8):2300029. doi: 10.1002/smsc.202300029 (PMC11935963; doi:10.1002/smsc.202300029)
Supplement: Supplementary file 1 — Supplementary Material [file SMSC-3-2300029-s001.pdf]

Sensing dynamically evolved short-range nanomechanical forces in fast-mutating single viral spike proteins

Amir Farokh Payam[a,b,*], Riccardo Funari[c,d,*], Gaetano Scamarcio[c,d], Nikhil Bhalla[a,b,*]

[a]*Nanotechnology and Integrated Bioengineering Centre (NIBEC), School of Engineering, Ulster University, Jordanstown, Shore Road, Northern Ireland BT37 0QB, United Kingdom*

[b]*Healthcare Technology Hub, Ulster University, Jordanstown, Shore Road, Northern Ireland BT37 0QB, United Kingdom*

[c]*Dipartimento Interuniversitario di Fisica, Università degli studi di Bari Aldo Moro, via Orabona 4, 70126 Bari, Italy*

[d]*Institute for Photonics and Nanotechnologies, CNR, Via Amendola, 173, Bari, 70125, Italy*

[*]Corresponding Authors: a.farokh-payam@ulster.ac.uk, riccardo.funari@uniba.it, n.bhalla@ulster.ac.uk

Reconstruction of the interaction force

For tapping force microscopy or in otherwards, amplitude modulation AFM, according to the definition of virial theorem, over one period of oscillation the following equations relating the AFM observables to the tip-surface force parameters can be obtained by [1]:

$$V_{ts} = \frac{1}{T}\int_0^{T_0} F_{ts}\big(z(t), \dot{z}(t)\big)z(t)dt = -\frac{kAA_0}{2Q}\cos(\phi) \quad \textbf{(S1)}$$

where $z(t) = Acos(\omega t + \phi)$ is the cantilever motion signal, A and ϕ are amplitude and phase of signal, k and Q are spring constant and quality factor of cantilever, respectively. F_{ts} is the interaction force and A_0 is the free amplitude. Figure S1 describe the distances and observables in tapping mode AFM.

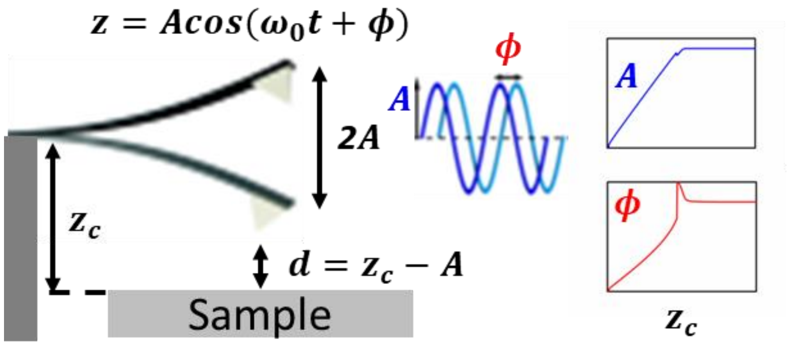

Figure S1. Schematic of the definition of distances and observables in tapping mode AFM. The amplitude and phase shift are the used to reconstruct the force curve. z is the instantaneous tip deflection; z_c is the probe–surface separation; d is the closest approach of the tip to the sample surface (it has either positive or negative values).

Following definitions in figure S1, V_{ts} can be described as [2]:

$$V_{ts} = \frac{1}{\pi k}\int_{z_c-A}^{z_c+A} F_{ts}\big(z(t), \dot{z}(t)\big)\frac{z-z_c}{\sqrt{A^2-(z-z_c)^2}}dz \quad \textbf{(S2)}$$

By applying the transformation $d = z_c - A$ where d is the closest tip sample distance to equation (S2), equation (S2) is converted to:

$$V_{ts} = \frac{2}{\pi k} \int_{d}^{d+2A} F_{ts} \frac{z-d-A}{\sqrt{A^2-(z-d-A)^2}} dz \quad \textbf{(S3)}$$

Then, by defining new variable u as:

$$u = \frac{z-d-A}{A} \quad \textbf{(S4)}$$

Equation (S3) is converted to:

$$V_{ts} = \frac{A}{\pi k} \int_{-1}^{1} F_{ts} \frac{u}{\sqrt{1-u^2}} du = -\frac{kAA_0}{2Q} cos(\phi) \quad \textbf{(S5)}$$

Utilizing the definition of Laplace transform of the force [3] as:

$$F_{ts}(d) = \int_0^{\infty} F_{ts}(\lambda) \exp(-\lambda)\, d\lambda \quad \textbf{(S6)}$$

Equation (S5) is solved by [4]:

$$\int_{-1}^{1} F_{ts} \frac{u}{\sqrt{1-u^2}} du = -\pi \int_0^{\infty} F_{ts}(\lambda) T(\lambda A) \exp(-\lambda d)\, d\lambda = \frac{kA_0}{2Q} cos(\phi) \quad \textbf{(S7)}$$

where $T(\lambda A)$ can be expressed in terms of the modified Bessel function of the first kind of order one $I_1(\lambda A)$ as:

$$T(\lambda A) = I_1(\lambda A) \exp(-\lambda A) \quad \textbf{(S8)}$$

Following our proposed method which was explained with detail in [4], using Pade approximation, inverse Laplace transform as well as fractional integrals and derivatives, following equation for force is extracted:

$$F_c(d) = 2k \int_d^{\infty} X dx + 2k \int_d^{\infty} \frac{\sqrt{A}}{8\sqrt{\pi(x-d)}} X dx - 2k \frac{\partial}{\partial d} \int_d^{\infty} \frac{A^{\frac{3}{2}}}{\sqrt{2(x-d)}} X dx \quad \textbf{(S9)}$$

where

$$X = \frac{A_0}{2QA} cos\phi \quad \textbf{(S10)}$$

From the reconstructed force curve, the adhesion force, stiffness and Young's modulus are extracted [2], [5], [6].

Energy Dissipation calculation

Considering the steady state motion of vibrating cantilever, the interaction between tip and sample surface is composed of both conservative and dissipative (non-conservative) interactions. So, the external mechanical energy supplied by the driving force to the cantilever (W_{ext}) is converted to hydrodynamic damping in the medium of interaction (W_{med}) and energy transferred to the sample (E_{diss}). As we assume the sinusoidal motion for the cantilever, the energy dissipated (per cycle) on the sample surface can be calculated by following equation:

$$E_{diss} = W_{ext} - W_{med} \quad \textbf{(S11)}$$

where

$$W_{ext} = \frac{1}{T}\int_0^T F_0 \cos(\omega t)\, \dot{z}(t) dt \quad \textbf{(S12)}$$

$$W_{med} = \frac{k}{TQ\omega}\int_0^T \dot{z}(t)^2 dt \quad \textbf{(S13)}$$

Replacing equations (S12) and (S13) in (S11) and solve for $z(t) = Acos(\omega t + \phi)$ gives following equation:

$$E_{diss} = \frac{\pi k A^2}{Q}\left(\frac{A_0}{A} sin\phi - 1\right) \quad \textbf{(S14)}$$

Calculation of Hamaker constant

To calculate Hamaker constant, by assuming that in most AM-AFM measurement the amplitude is significantly larger than the range of the interaction occurring between tip and surface, especially in the present case in which we focus on attractive regime of short range interaction, and knowing that the cantilever has sinusoidal motion, for the equation (S3), the following conversion at $z \to d$ is acceptable [7]:

$$\frac{z-d-A}{\sqrt{A^2-(z-d-A)^2}} \approx -\sqrt{\frac{A}{2(z-d)}} \qquad \textbf{(S15)}$$

This leads to:

$$V_{ts} = \frac{\sqrt{2A}}{\pi k}\int_{D}^{D+2A} \frac{F_{ts}}{\sqrt{z-D}}dz \qquad \textbf{(S16)}$$

Applying (S16) in (S1), the following equation is obtained:

$$\frac{1}{\pi}\int_{D}^{D+2A} \frac{F_{ts}}{\sqrt{z-D}}dz = \frac{k^2 A_0 A^{1/2} cos\phi}{Q\sqrt{2}} \qquad \textbf{(S17)}$$

Hence, the force is obtained as:

$$F_{ts}(D) = -\frac{\partial}{\partial D}\int_{D}^{D+2A} \frac{k^2 A_0 A^{1/2} cos\phi}{Q\sqrt{2}\sqrt{z-D}}dz \qquad \textbf{(S18)}$$

Considering the van der Waals force equation as [8], [9]:

$$F_{vdw} = -\frac{HR}{6d^2} \qquad \textbf{(S19)}$$

Where H is Hamaker constant, equating equations (S18) and (S19) the following approximated equation for the Hamaker constant can be obtained:

$$H \approx \frac{3kA_0A^2\cos(\phi)}{QR}\left[\left(\frac{d+A}{A}\right)^2 - 1\right]^{3/2} \quad \textbf{(S20)}$$

Topography and phase imaging of spike proteins

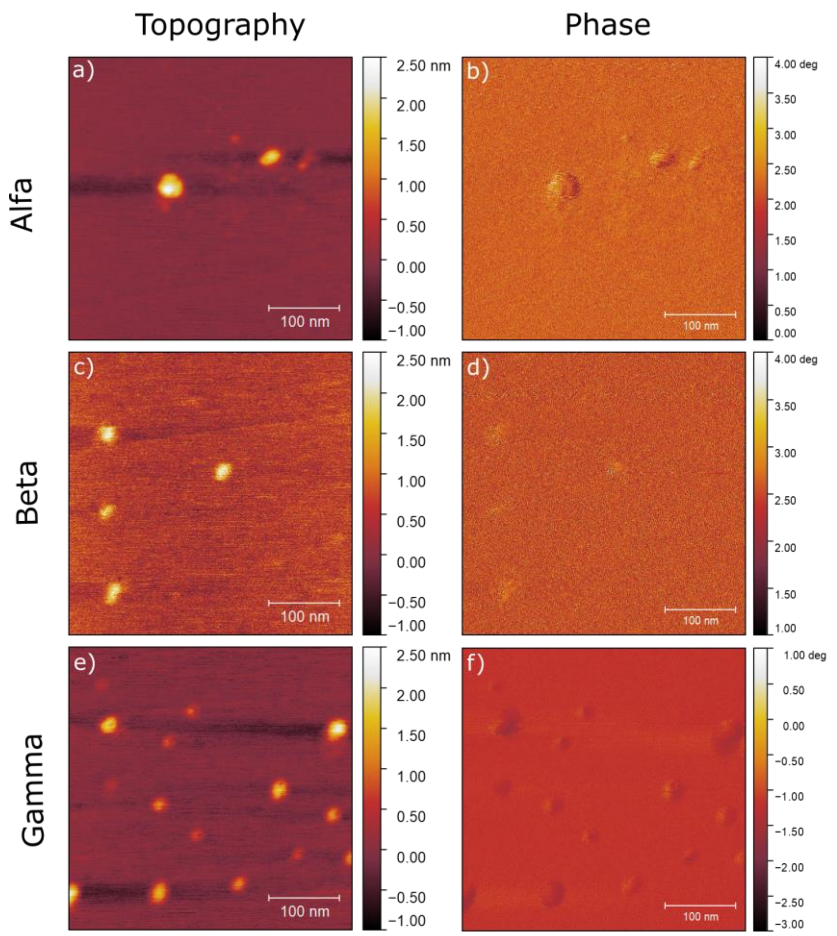

Figure S2. *Height (left panels) and phase (right panels) imaging of Alpha (a, b), Beta (c, d) and Gamma (e, f) Spike proteins on freshly cleaved mica by AM-AFM.*

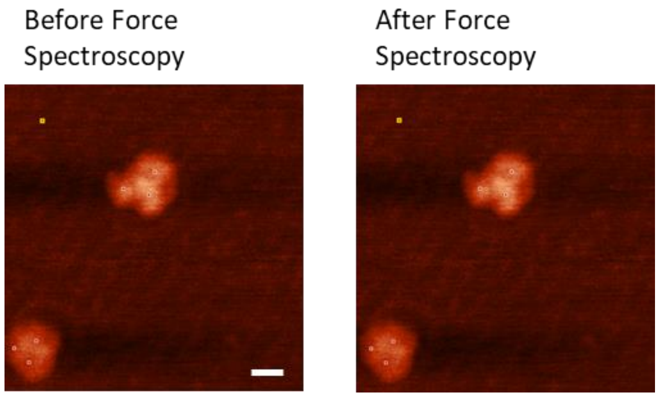

Figure S3. *AFM topography recorded before and after force spectroscopy measurements. The figures show that there are negligible drift in the sample position, and protein shapes remain*

consistent after the compression caused by the contact with the probe. The squares highlight the position where the amplitude-distance curves are collected. The white bar corresponds to 20 nm.

We also summarize the mechanical properties of the proteins with the mutation Alpha, Beta and Gamma spike proteins reported in the literature, see figure S4a to c. Here essentially, we show E, k, F_{ad} and H as i, ii, iii and iv in the subfigures S4a, S4b and S4c. From, figure S4a, see that there is more variation in the E of Alpha compared to other k, F_{ad} and H of Alpha. For Beta and Gamma, Hamaker constant varies more in comparison to the other 3 parameter. These difference and variation in the individual mechanical property can be associated with all mutations listed in the figure S4a to c.

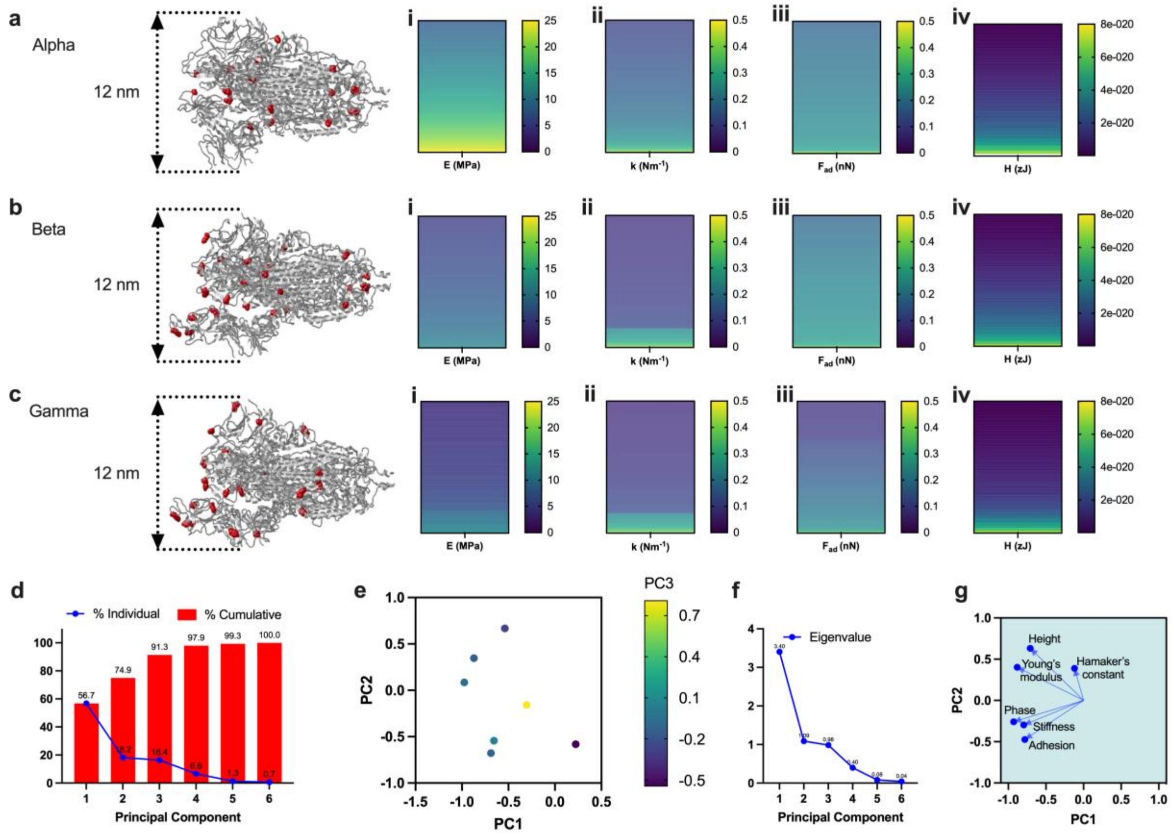

h

	N-terminal domain mutations	Receptor-binding domain mutations	Other spike mutations

**Alpha**	Δ 69-70, Δ144	N501Y	A570D, D614G, P681H, T716L, S982A and D1118A
**Beta**	Δ 242-244, R246L, D215G, D80A, L18F	K417N/T, E484K, N501Y	D614G, A701V
**Gamma**		K417N/T, E484K, N501Y	D614G

Figure S4. Statistical analysis *a), b) and c) show the Cryo-EM structures(PDB codes 7EDH, 7LYN and 7M8K for Alpha, Beta and Gamma, respectively) and heat maps demonstrating variations in Young modulus (E), Stiffness (k), Adhesion (F_{ad}) and Hamaker constant (H) of Alpha, Beta and Gamma proteins respectively. The positions of the mutations on the 3D structures are highlighted in red. d) to g) is principal component analysis (PCA) of six variables, E, k, F_{ad}, H, phase and height of the protein imaged using atomic force microscope. Specifically, d) shows the contribution of each principal component (PC) to the variance in the dataset; e) multiple variable plot showing the relationship between PC1, PC2 and PC3; f) shows eigenvalues associated with PC; g) shows loading plot demonstrating the relationship between PC1 vs PC2. h) list of characteristic mutations for Alpha, Beta and Gamma.*

Note that we do not associate a particular mutation with a given mechanical property and therefore the heat maps in the figure S4a to c should be only used as qualitative guide to an eye demonstrating differences among Alpha, Beta and Gamma proteins in relation to mutations. We have also extended our investigation to perform principal components analysis (PCA) to find relationship between height and phase obtained from a typical AFM scan and *E, k*, F_{ad}, and *H* of the protein. Figure S4d shows cumulative variance represented by all principal components (PC). For data analysis, we choose 3 PC-PC1, PC2 and PC3 as they represent more than 90% of the variance within the data. The relationship between PC1, PC2 and PC3 is shown in figure S4e while eigenvalues associated with all six PCs is given in figure S4f. Figure S4g

shows the exclusive relationship between PC1 and PC2 based on Kaiser rule criteria, which chooses only those PCs for comparison which have eigenvalues are greater than 1. The resulting PC1 *vs.* PC2 shown in figure S4g is called as a loadings plot which shows the relationship between all six variables by plotting vectors. The closeness of phase vector to the stiffness and adhesion suggests that phase changes are mainly contributed by the stiffness and adhesive forces exerted by the protein on the tip of the AFM cantilever which was explained already in our previous works [2], [10] as well as demonstrate the significant contribution of adhesion hysteresis in the phase contrast and subsequently dissipation of spike proteins. Similarly, the Hamaker constant can be associated to the Young's modulus which is also closely associated with the measured height of the protein. This is in agreement with our recent findings which emphasize the importance and contribution of Hamaker constant in the calculation/modeling Young's modulus in AFM measurement especially for soft matter [2]. Even though a fundamental relationship can be established between different parameters using our PCA, such between H and E, the PCA here should only be used for qualitative analysis. This is because PCA establishing relationships based on the principles of linear regression whereas the relationships between different mechanical parameters investigated in our work is non-linear in nature.

PCA analysis methodology

The principal components, in figure S4, are chosen based on the classical method of Kaiser-Guttman rule (also known as the Kaiser criterion) to select principal components (PC). For PCA, we have considered the following variables: height and phase obtained from a typical AFM scan and E, k, F_{ad}, and H of the protein. Each of the principal components (linear combination/mixture of starting variables) is uncorrelated and the information from the initial variables is compressed into these *via* orthogonal eigenvectors that represent the maximal amount of variance in dataset. Thus, the PCA essentially extracts the smallest number of

components that describe the most variation of the original dataset with minimal loss of information. In this method, we assume that with the standardized data, the variance of each of the original variables is equal to 1. Therefore, a PC with an eigenvalue greater than 1, contains more variance than a single variable in the original data. Additionally, we have also checked the percentage of total variance explained by each of the principal components and ensured that the selected components have at least 90% of the total variance in our original data (PC1, PC2 and PC3). All plots and analyses within the PCA and statistical evaluation of the data are performed using GraphPad Prism 9 software.

References

[1] J. R. Lozano and R. Garcia, "Theory of phase spectroscopy in bimodal atomic force microscopy," *Phys. Rev. B - Condens. Matter Mater. Phys.*, vol. 79, no. 1, p. 014110, Jan. 2009.

[2] A. F. Payam, A. Morelli, and P. Lemoine, "Multiparametric analytical quantification of materials at nanoscale in tapping force microscopy," *Appl. Surf. Sci.*, vol. 536, no. July 2020, p. 147698, 2021.

[3] J. E. Sader and S. P. Jarvis, "Accurate formulas for interaction force and energy in frequency modulation force spectroscopy," *Appl. Phys. Lett.*, vol. 84, no. 10, pp. 1801–1803, 2004.

[4] A. F. Payam, D. Martin-Jimenez, and R. Garcia, "Force reconstruction from tapping mode force microscopy experiments," *Nanotechnology*, vol. 26, no. 18, p. 185706, May 2015.

[5] J. Shang *et al.*, "Cell entry mechanisms of SARS-CoV-2," *Proc. Natl. Acad. Sci. U. S. A.*, vol. 117, no. 21, 2020.

[6] J. Tamayo, "Energy dissipation in tapping-mode scanning force microscopy with low quality factors," *Appl. Phys. Lett.*, vol. 75, no. 22, pp. 3569–3571, 1999.

[7] H. Hölscher and U. D. Schwarz, "Theory of amplitude modulation atomic force microscopy with and without Q-Control," *Int. J. Non. Linear. Mech.*, vol. 42, no. 4, pp. 608–625, 2007.

[8] S. Kawai *et al.*, "Van der Waals interactions and the limits of isolated atom models at interfaces," *Nat. Commun.*, vol. 7, no. May, pp. 1–7, 2016.

[9] E. T. Herruzo and R. Garcia, "Theoretical study of the frequency shift in bimodal FM-AFM by fractional calculus," *Beilstein J. Nanotechnol.*, vol. 3, no. 1, pp. 198–206, 2012.

[10] A. F. Payam, J. R. Ramos, and R. Garcia, "Molecular and nanoscale compositional contrast of soft matter in liquid: Interplay between elastic and dissipative interactions," *ACS Nano*, vol. 6, no. 6, pp. 4663–4670, 2012.
